# Review about the Application of Fractal Theory in the Research of Packaging Materials

**DOI:** 10.3390/ma14040860

**Published:** 2021-02-11

**Authors:** Qingshan Duan, Jiejie An, Hanling Mao, Dongwu Liang, Hao Li, Shuangfei Wang, Chongxing Huang

**Affiliations:** 1 School of Light Industry and Food Engineering, Guangxi University, Nanning 530004, China; qs_duan@gxu.edu.cn (Q.D.); anan@st.gxu.edu.cn (J.A.); liangdongwu@gxu.edu.cn (D.L.); LH020900@163.com (H.L.); wangsf@gxu.edu.cn (S.W.); 2 Guangxi Key Laboratory of Clean Pulp & Papermaking and Pollution Control, Nanning 530004, China; 3 School of Mechanical Engineering, Guangxi University, Nanning 530004, China; maohl79@gxu.edu.cn

**Keywords:** fractal theory, fractal dimension, packaging materials, property, preparation

## Abstract

The work is intended to summarize the recent progress in the work of fractal theory in packaging material to provide important insights into applied research on fractal in packaging materials. The fractal analysis methods employed for inorganic materials such as metal alloys and ceramics, polymers, and their composites are reviewed from the aspects of fractal feature extraction and fractal dimension calculation methods. Through the fractal dimension of packaging materials and the fractal in their preparation process, the relationship between the fractal characteristic parameters and the properties of packaging materials is discussed. The fractal analysis method can qualitatively and quantitatively characterize the fractal characteristics, microstructure, and properties of a large number of various types of packaging materials. The method of using fractal theory to probe the preparation and properties of packaging materials is universal; the relationship between the properties of packaging materials and fractal dimension will be a critical trend of fractal theory in the research on properties of packaging materials.

## 1. Introduction

Mandelbrot proposed the concept of fractal in 1975, which refers to the graph, phenomenon, or process with self-similarity [1]. Fractal objects generally exist in nature, such as curved coastline, rough surface, and so on [2,3]. At present, fractal theory has been extensively used in natural science, engineering technology, social culture, and other fields [4,5,6,7]. In the packaging industry, the surface morphologies of all kinds of packaging materials are complex and irregular, but they have features of self-similarity, which is highly appropriate for analysis by fractal theory. Many scholars have been using fractal theory to compute the fractal dimension of packaging materials and build the relationship between the properties of packaging materials and fractal dimension. As is known to all, a lot of materials can be availed in the packaging industry, so packaging materials involve a wide range of materials. In order to ensure people have a comprehensive and in-depth understanding of the fractal in a variety of packaging materials, it is necessary to summarize and sort out the application progress of fractal theory in the research of packaging materials, to promote the development of fractal theory in the research of packaging materials.

In this paper, the fractal analysis methods applied to inorganic materials such as metals and ceramics, polymers, and their composites are reviewed by the aspects of the basic theory of fractal and fractal feature extraction. Through the fractal dimension model of packaging materials, the relationship between the fractal characteristic parameters and the preparation process and properties of packaging materials is discussed, respectively. The purpose of the above is to present important insights into the fractal research of packaging materials.

## 2. Basic Theory of Fractal

### 2.1. The Concept of Fractal

In 1982, Mandelbrot established the mathematical definition of fractal. In Euclidean space, if the Hausdorff dimension of a set F is greater than its topological dimension, the set F is a fractal set [8]. Since the definition is not applicable in practical application, he later proposed that the shape the components of which resemble the whole to some extent are called fractal [9].

**Figure 1:**
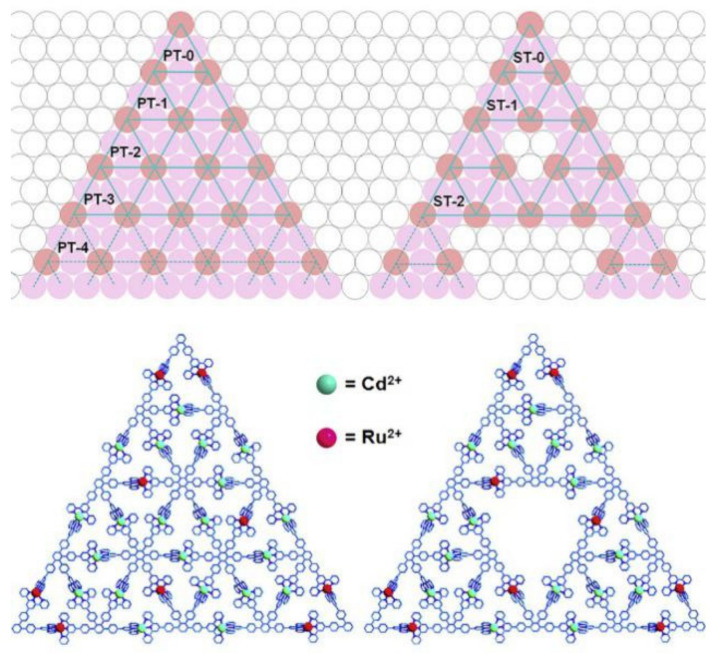
Sierpiński Triangular Fractals.

Fractal has features of self-similarity, scale invariant, and self-affine [10,11,12]. Self-similarity may be exactly similar to the Sierpiński triangle [13], as shown in Figure 1, the approximate similarity of branch bifurcation [14], or statistical similarity [15]; the first is called accurate or mathematical fractal, and the second and third type is known as random fractal [16]. The preparation process, surface morphology, and performance change process of various packaging materials have fractal features, which are fractal surfaces in statistics. Scale invariant is the property that the shape, irregularity, and complexity of an object will not change if it is enlarged or shrunk in any local area of the object. This object can have fractal features in a scale invariant range. Self-affine is a special case of self-similarity, which refers to the property that the proportion of transformation from local to global in diverse directions is not necessarily the same. The feature of fractal objects can be described by fractal dimension. If the fractal features are dissimilar in different scale invariant intervals, the object has multifractal features. In the measurement theory, when the measures studied are the same in unlike scales, or at least the same in statistics, it can be said that the measures studied are self-similar, which is called multifractal (MF). MF can be expressed by generalized dimension spectrum(q-Dq) or α-f(α) multifractal spectrum [17].

### 2.2. Fractal Feature Extraction and Fractal Dimension Calculation

When fractal dimension is utilized to represent the characteristics of fractal objects, the fractal features of objects are extracted first, and then the fractal dimensions of objects are calculated. The normally used method to extract the fractal feature of packaging materials is to employ the material micromorphology in the preparation process, surface, or fracture morphology of packaging material, and the test data in the property change process of packaging material (e.g., acoustic emission (AE) signal [18]) to carry out image gray processing and noise reduction processing, respectively. Common methods for calculating fractal dimension include the box counting method [19], correlation dimension method [20], Hurst index method [21], slit island method [22], yard stick method [23], area-perimeter method [24], Sierpiński carpet method [25], semi-variance method [26], and power spectral density [27] (PSD) method. The main methods to obtain object image are scanning electron microscope [28] (SEM), transmission electron microscope [29] (TEM), atomic force microscope [30] (AFM), and other imaging techniques. When the object is an image, after gray processing, binary processing is carried out [31]. Finally, the fractal dimension of the object can be computed by a fractal dimension algorithm of graphics, for instance the slit island method, box counting method, and others. If the object is a signal, it is denoised before the fractal dimension is calculated by fractal dimension calculation methods for signals (e.g., PSD, correlation dimension and others). In addition, the fractal feature extraction of porous packaging materials can be obtained by mercury intrusion porosimetry [32] (MIP) and adsorption method [33], and then the fractal dimension can be acquired. The fractal dimension of packaging materials suitable for small-angle X-ray scattering [34] (SAXS) analysis can be obtained by the following method. In SAXS, the scattering intensity *I*(***l***) is exponentially related to the scattering vector ***l***:

(1) $$I\left(l\right)\propto l^{-\alpha },$$

When 1 < *α* < 3, the fractal dimension is described as mass fractal dimension Dm = *α*; when 3 < *α* < 4, the fractal dimension called surface fractal dimension Ds = 6 − *α*. The scatterers with surface fractal are more dense than those with mass fractal.

Many packaging materials have MF feature, and the application and innovation of MF algorithm [35] have become one of the important choices to analyze the performance of such packaging materials. For the description of non-uniform structures with rich scale features, in the mass fractal region, the box counting dimension of MF spectrum is consistent with the scattering index of the X-ray spectrometer curve, and changes from short-range mass fractal area to the long-range mass fractal region [36]. When MF evaluates and compares the heterogeneity of various porous samples, the pixel size of the sample must be the same, the sample volume must be larger than the representative volume element, the MF dimension should be normalized to the determined porosity value, and the influence of sub-resolution pores on small-scale should be ignored; the real samples can be divided into less and heterogeneous groups through the normalized fractal dimension [37]. The packaging materials of different structures and properties with MF characteristics should be treated differently. Xi et al. [38] proposed two-dimensional multifractal cross-correlation analysis based on the partition function (2D-MFXPF), two-dimensional multifractal cross-correlation analysis based on the detrended fluctuation analysis (2D-MFXDFA), and two-dimensional multifractal cross-correlation analysis based on the detrended moving average analysis (2D-MFXDMA), and compared their differences in two-dimensional multiplicative cascades. Finally, the 2D-MFXDFA method was made available to real images to reveal the interesting MFs in the cross correlation of material structure. The research offered a valuable reference for the potential application of MF algorithm in SAR image classification and detection. Kovalenko et al. [39] constructed a new gradient pixel fractal analysis method, which is particularly appropriate for establishing the MF feature of multi-scale composite inclusions in materials and nanostructures, so as to connect the indexes of structure and phase inhomogeneity with the changes of physical and chemical properties during the development of new material. The above researches provided more in-depth and detailed references for people to understand the property of packaging materials with MF feature, and more advanced ways for researchers.

## 3. Properties and Fractal Analysis of Metals and Ceramics

### 3.1. Fractal and Properties Analysis of Metal Packaging Materials

Metal is one of the prevalently used packaging materials. During processing and service, metal experiences complex physical and chemical changes. Due to the nonlinear feature of these processes, the surface morphology of metal is very intricate, which often has statistical self-similarity within a certain scale range, that is, the surface morphology of metal packaging materials has fractal characteristics. [40] Fractal method can be implemented to texture recognition on the copper surface [41], and the accuracy of surface texture quality can be fully quantified by analyzing copper surface texture with Hausdorf dimension [42]. Later, a robust numerical model was created to predict rough contact parameters by the inherent characteristics of the surface micro geometry, i.e., the measurement invariants. The rough surface of metal could be described as a self-affine fractal, which can be expressed by the second moment of its power spectrum [43].

The size and morphology of nano metal particles in the composites also have fractal characteristics. Lashgari et al. [44] computed the fractal dimensions of 30 SEM images of Mn-Cr bimetallic nanocomposites by using the self-similarity principle and box counting method. It was noted that the fractal dimensions were scattered within the scope of 1.60 ~ 1.98, and the distribution was uneven. This indicated that the size and morphology of nanoparticles in the composite were different.

The surface morphology and property change process of metal packaging materials have fractal characteristics when their properties deteriorate and fail. The factors affecting the fractal dimension of material fracture morphology mainly include the generation conditions of fracture surface, the extraction method of fractal fracture, and the calculation method of fractal dimension of fracture morphology; since box counting dimension is simple and easy to achieve, it is preferred to obtain the fractal dimension of fracture morphology [45]. However, the accuracy of the box counting dimension is not high, and other methods such as correlation dimension and Hurst index are typically utilized. A recent example is the use of mutual information and chaos method to reconstruct the time series of electrochemical potential noise of 304 and 316 L stainless steel under three conditions of passivation, localized, and uniform corrosion [46]; the correlation dimension of local corrosion of stainless steel is higher than that of passivation. Phase space reconstruction supplied a new method for studying electrochemical noise signal and some fresh information for identifying corrosion types [47]. When the Hurst index is utilized to analyze the defect characteristics on the fracture surface of aluminum alloy under dynamic loading, the Hurst index of the fracture surface of aluminum alloy under the high-speed loading is more uniform. Under low speed loading, the Hurst index of localized fracture is smaller, but Hurst index is larger when the defect is caused by localized fracture and coarsening of crystal particles in the defect structure [48]. When the crack length exceeds a critical value, the crack will propagate rapidly at high speed [49]. Under alternating loading, the fracture morphology of AlMg6 is MF, the fish-eye cracks in the fatigue fracture zone are formed owing to the qualitative change of the initial cracks in the process of fatigue accumulation, and in the final stage of crack growth and fracture, the material damage fractal characteristics transform from single fractal to multi fractal [50]. By studying the similarity of the dynamic failure process of metal with distinct geometry shapes under the exterior impact with diverse amplitude-time characteristics, fractal dimension may quantify the damage of materials. The self-similar behavior under a high-strength external impact makes it possible to determine the dimensionless ratio. By using nonlinear physics and fractal theory to quantify the characteristics of dissipative structure, the analogous metal dispersion process can be established; this similarity is caused by its pure non-equilibrium dissipation such as scale invariance [51]. The above research shows that the property change or failure of metal packaging material can be characterized by its fractal dimension, which could be obtained by box counting dimension, correlation dimension, and Hurst index; material damage has the characteristics of transition from single fractal to MF, which can be quantified by fractal dimension, and MF analysis is a relatively new research direction on the property’s analysis of metal packaging materials.

**Figure 2:**
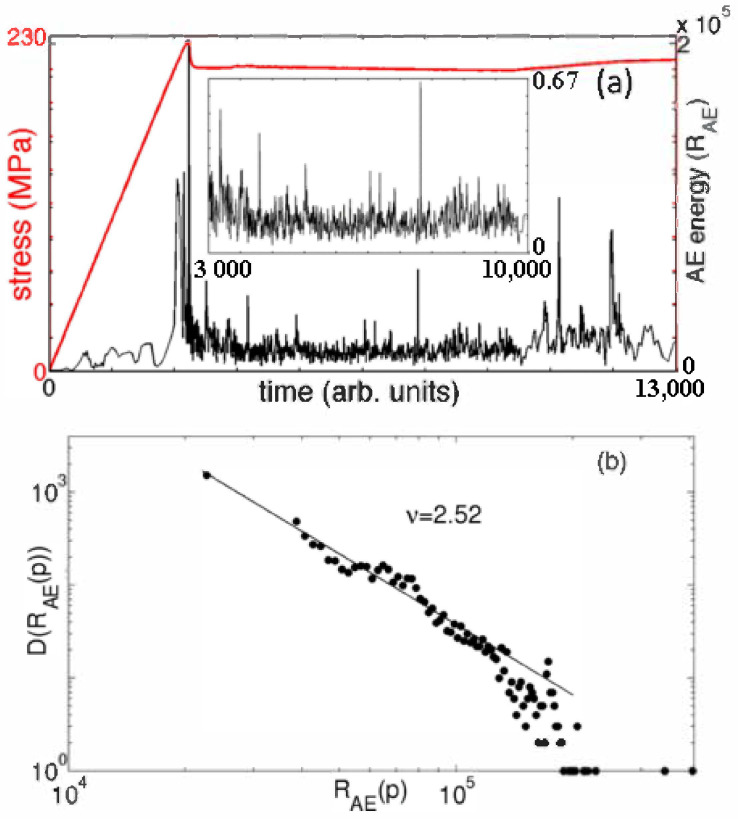
Model acoustic energy for the Lüders-like propagating band along with the stress-strain curve (**a**), and corresponding power-law distribution for AE signals (**b**).

**Figure 3:**
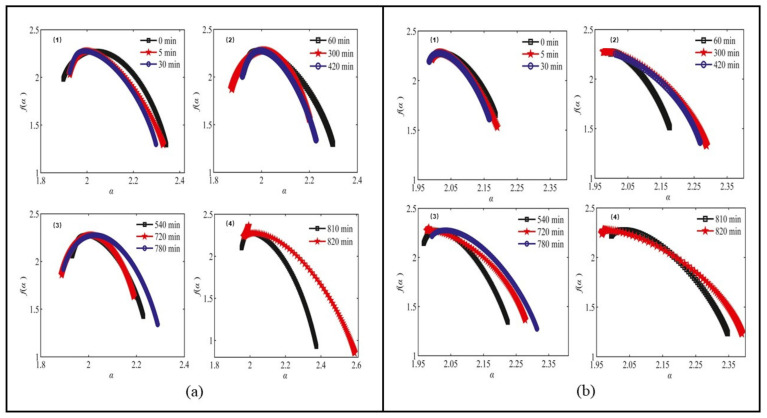
Multifractal spectrum surfaces (**a**) and ring surfaces (**b**) during the wear process.

The MF dimension applies equally to the study of the properties of metal materials. Kumar et al. [52] proposed a general framework for calculating AE signals, which is used for alloy materials Portevin-Le Chatelier band [53] and lüders-like band [54], as displayed in Figure 2. Through the MF spectrum analysis, it is shown that the fractal dimension of these AE signals increases with the increase in strain rate. Laser confocal microscope (LSCM) is fit for obtaining the three-dimensional morphology of the material. By measuring the three-dimensional morphology of the disc and ring of GCr15 steel and H70 brass, the MF spectrum of the worn surface is obtained by box counting method, as shown in Figure 3. According to the variation characteristics of the spectrum width Δα and spectrum difference Δf(α) of the MF spectrum, it is shown that the worn surfaces at dissimilar wear stages have different MF characteristics, revealing the singularity and nonlinear behavior of the friction process [55]. The MF method cannot only characterize the mechanical properties of metal, but also effectively predicts its mechanical properties. Based on the MF Renyi spectrum [56], Krovikov et al. [57] tested the sensitivity of ultimate strength of cast iron to carbide information size, final bending strength to carbide fractal size, impact strength to carbide-related size, and hardness to graphite fractal size. Based on the analysis of microstructure elements such as carbide and graphite, a fractal model of cast iron quality characteristics was put in place to evaluate and predict the mechanical properties of cast iron. The above research shows that multifractal dimension is a new method that can be used to evaluate or even predict the mechanical properties of metal materials. It is expected to be adopted to analyze and predict the mechanical properties of more kinds of metal materials or containers during circulation and use in the packaging field.

### 3.2. Fractal and Property Analysis of Ceramics

The surface microstructure of ceramics has fractal characteristics. Using MF spectrum function and generalized Renyi spectrum to analyze the surface microstructure of ceramic materials, we can judge the homogeneity of surface structure and its formation conditions [58].

**Figure 4:**
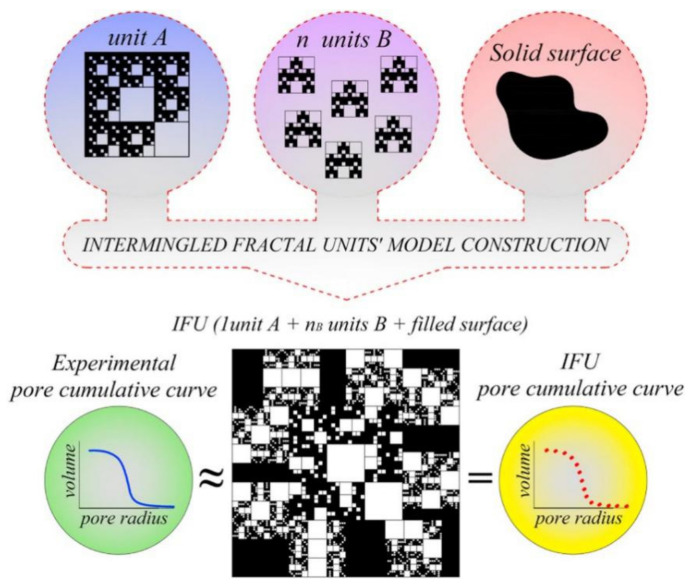
IFU procedure to simulate pore accumulation curve.

**Figure 5:**
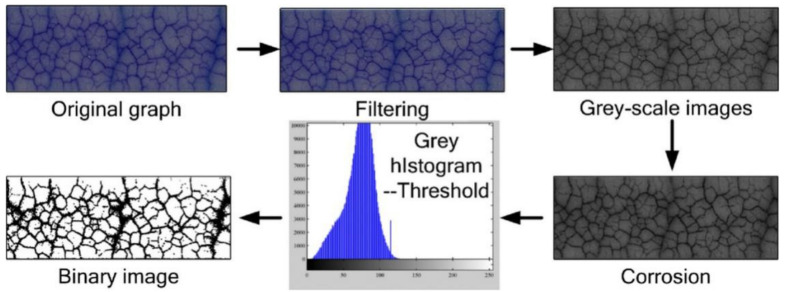
Image preprocessing method based on original crack patterns.

Porous ceramics have characteristics of large specific surface area, filtration, adsorption, and heat insulation due to its porous structure. Porous ceramic can be regarded as a porous structure composed of three different phases A, B, and C. The Sierpiński blanket is defined as three parts of A, B, and solid phase C by IFU method, and the pore size distribution is calculated, as indicated by Figure 4. Then, the bending strength is analyzed and computed by combining the model with the classical structural mechanics’ theory [59]. The method of combining experimental evidence and fractal geometry can be adopted to study crack propagation under thermal shock. The box counting method is used to obtain the fractal dimension of crack propagation on the inner surface (Figure 5) and cooling surface of alumina ceramics under thermal shock condition. Under various thermal shock temperatures, the fractal dimension is highly sensitive to the change of cracks. The smaller the particle size is, the higher the fractal dimension of crack morphology is. The fracture energy of microcracks with fractal in quasi brittle solids can explain the relationship between crack length and crack length. When the crack length is identical but the fractal dimension is larger, the fracture energy absorbed by the crack is greater [60]. Vukovic et al. [61] described a new method for analyzing the microstructure properties of materials, which was used to predict the final properties of ceramics by fractal specialty programming in a fractal field simulator generating structure, grains, and pores.

In conclusion, ceramics are brittle and easy to be damaged in the process of storage and transportation. The research of fractal theory in ceramics mainly focuses on the relationship between structure and mechanical properties and its property prediction; fractal research on other aspects of porous ceramic materials, such as filtration, adsorption, heat insulation, and other properties is less, which could become one of the important directions of follow-up research.

## 4. Properties and Fractal of Polymers and Their Composites in the Packaging Field

Polymers and their composites are widely used in the field of packaging materials, including plant fiber, petroleum-based, and bio-based polymer materials, and composite materials with the above components. In this section, the research statuses of fractal characteristics, material preparation and properties, and fractal dimension models of various types of materials are reviewed.

### 4.1. Fractal in the Preparation of Polymers and Their Composites in the Packaging Field

**Figure 6:**
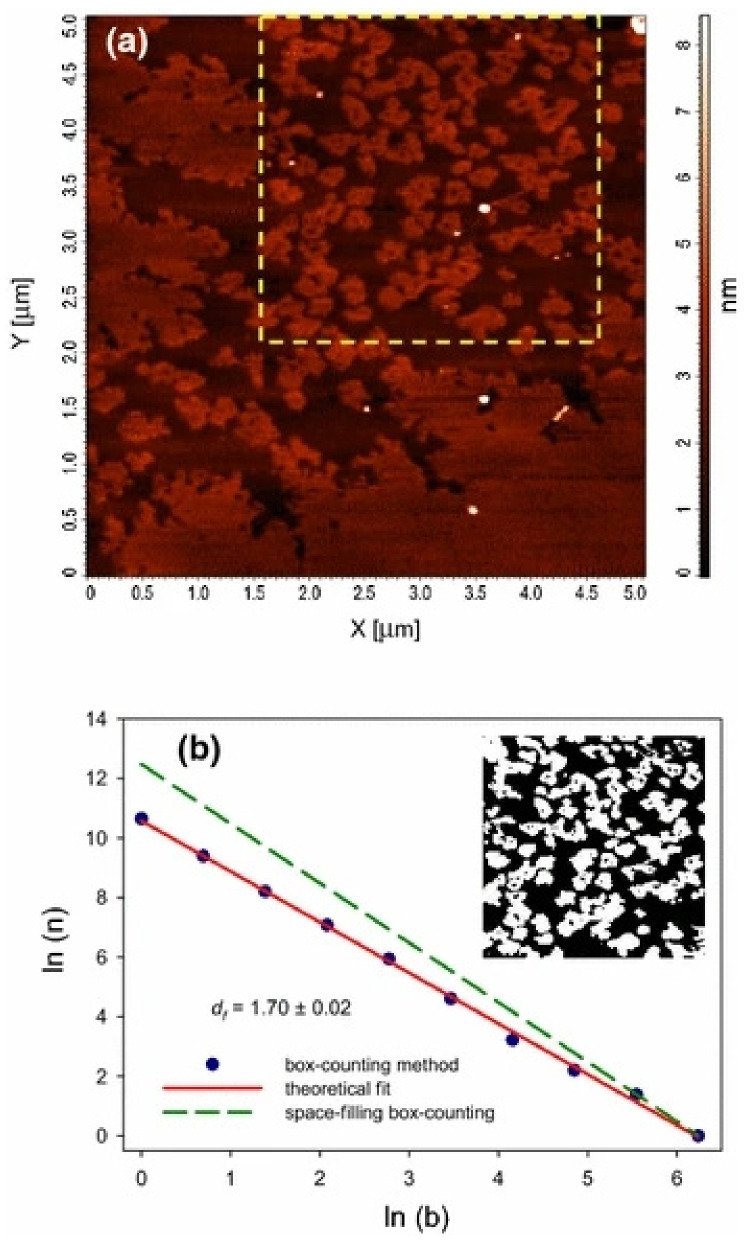
An AFM image (**a**) and its fractal dimension (**b**) of a rosette-shaped fractal structure limited by a plain region.

**Figure 7:**
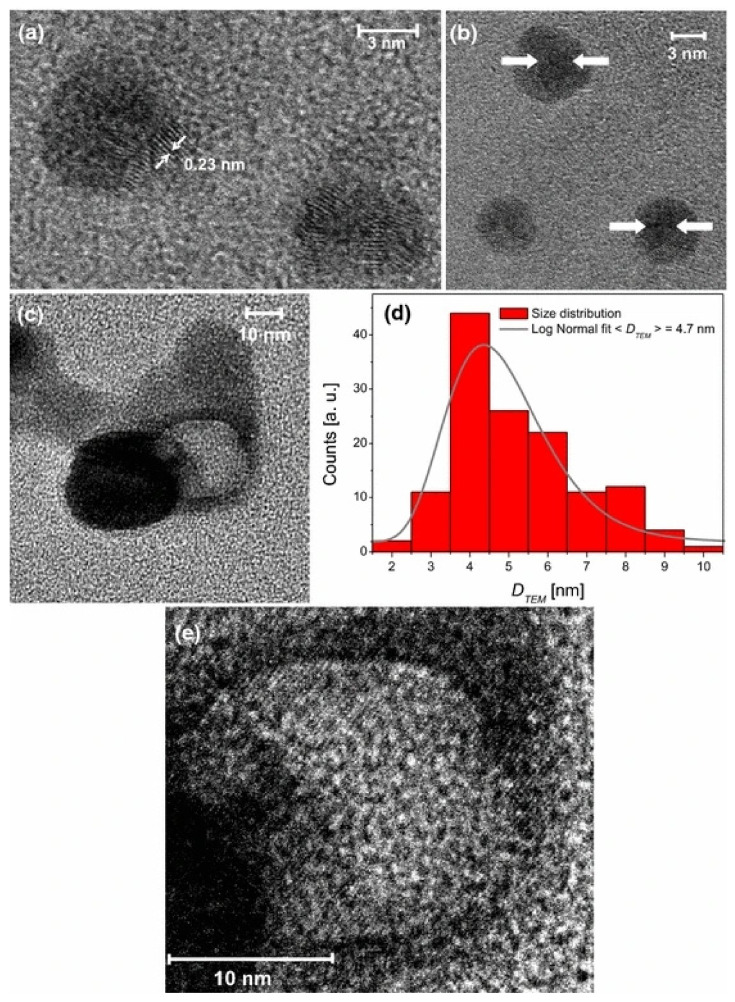
TEM images of Nps present in the colloidal suspension generated by femtosecond laser ablation: (**a**) isolated silver bare core Nps, (**b**) isolated silver core–shell Nps, (**c**) hollow metal Np together with a bare core Np, (**d**) size histogram obtained by measuring 140 particles within the sample and log-normal fit curve, and (**e**) enlargement of panel c where Bragg planes in the central zone of the hollow Np can be seen.

In the preparation process of polymers and their composites, the preparation environment, and the formation of crystals and aggregates have fractal characteristics. For obtaining the relationship between gas-liquid interfacial tension and its fractal dimension, Wang et al. [62] put forward a method to calculate the fractal dimension of gas-liquid interface. In the suspension, the fractal dimension of different systems is different; under shear, the suspension structure may be more inclined to reaction limited cluster-cluster aggregation(RLCA), and a more flocculated structure or particle rearrangement may exist in the suspension, which can result in a dense filler structure in the shear suspension structure [63]. As for the nanoparticles doped in polymer materials, the diffusion of nanoparticles in fractal interior and surface determines the shape of islands left on the surface after fractal fragmentation [64]. Santillán et al. [65] reported the self-assembled fractal structure of spherical Ag nanoparticles (NPS) prepared in water, such as Figure 6. In the prepared colloidal suspension, the fractal aggregates with size of about 31 nm were observed, as shown in Figure 7. In order to understand the growth mechanism of nano agglomerates and reveal the correlation between microstructure and macroscopic properties, Liu et al. [66] tested and analyzed the multifractal structures of pure polyimide (PI) film and inorganic nano hybrid PI film, and studied the spatial distribution and mass uniformity of the scatterers.

The materials prepared by self-assembly technology have fractal characteristics. In the process of preparing layer-by-layer self-assembled antibacterial films of hyaluronic acid (HA) and chitosan (CHI) biopolymers at two pH values (5 and 3), the fractal dimension of the pH 5 series was about 2.2, and the irregularity of the initial random adsorption process was the least. When the pH value was reduced to 3, the fractal dimension increased to 2.5, indicating that the random adsorption process was in the transition of diffusion limited aggregation [66]. In the electrospinning process, the length of the electrospinning needle is controlled to realize the structural control of materials at the molecular scale, which shows that the hierarchical structure of macromolecules in the self-assembly process has fractal characteristics [67]. Fractal patterns can be formed in the process of chemical deposition; the main factors affecting the formation rate and final geometry of aggregation fractal patterns are the molecular weight of polymers, the selection of reaction electrodes, and air exposure [68].

To sum up, in the preparation of polymers and their composites in the packaging field, we can analyze and predict the properties of materials via the fractal characteristics of diffusion of nano-particles in polymer materials, the growth mechanism of nano aggregates in solution systems, and the fractal characteristics of self-assembly technology, so as to obtain ideal polymer materials and their composites for packaging. At the same time, the self-assembly technology forming fractal pattern can be applied to obtain an ideal material surface.

### 4.2. Fractal Characteristics and Fractal Dimension of Polymers and Their Composites in the Packaging Field

**Figure 8:**
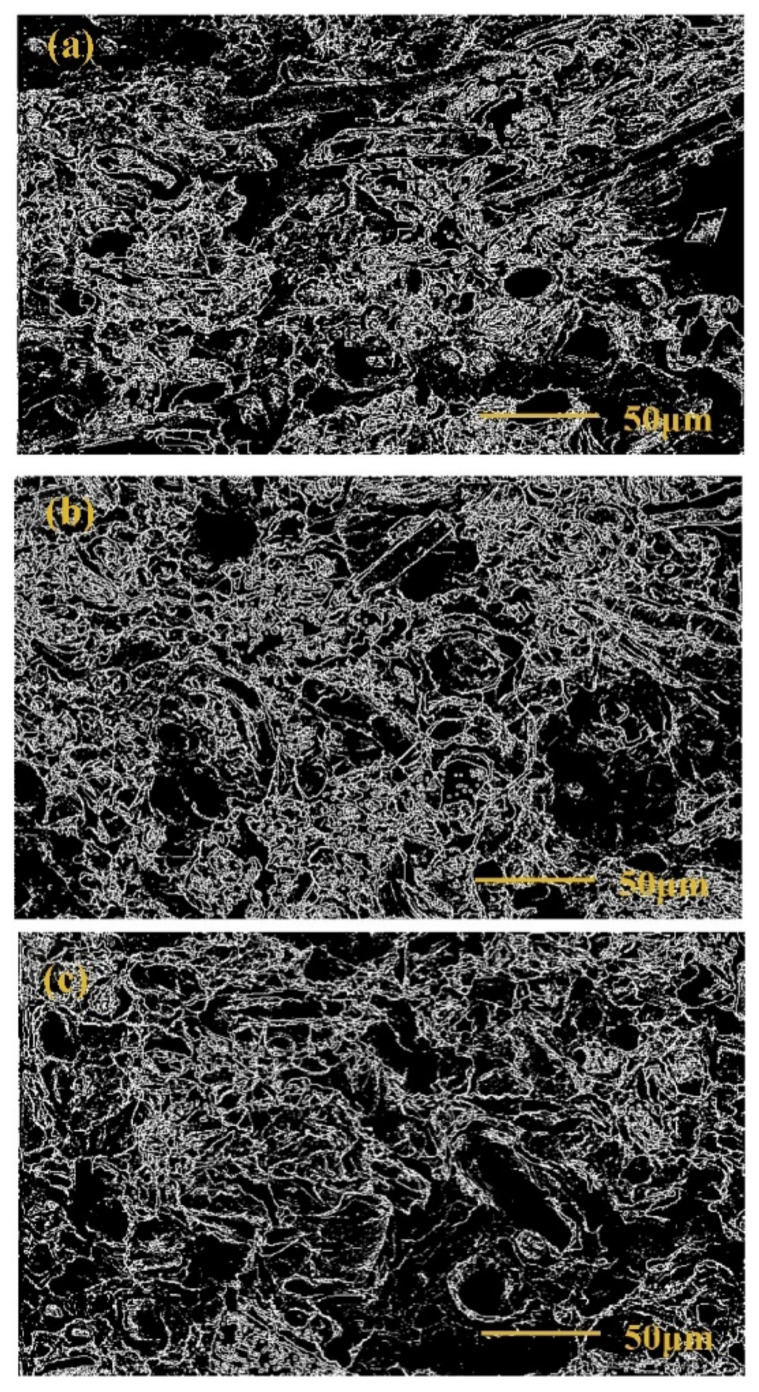
Binary pictures of composites fracture surface for EMP (**a**), IMP (**b**), and HPMP (**c**).

Searching for the characteristics and laws of polymer and their composites surface and component dispersion, numerous studies use different methods to characterize fractal characteristics and fractal dimensions. Using a microscope, Ritter et al. [69] discovered that the growth of cyclic fatigue crack in the epoxy resin/glass interface region presented viscosity and fractal. Nie et al. [70] calculated the fractal dimension of TEM images of SiO_2_/acrylate composites, and found that the closer the fractal dimension is to 2, the better the dispersion uniformity of the dispersed phase is. Zhang et al. [71] found that the longitudinal shear strength of wood has a positive correlation with the fractal dimension of fracture surface, while transverse shear strength has a linear positive correlation with it. For studying the relationship between interfacial adhesion and preparation technology, Nano calcium carbonate impregnation modification (IM) is employed as an effective way to improve the interface interaction of the bamboo fiber reinforced HDPE composites. Fractal theory and dynamic mechanics were used to analyze the composites produced by three processes: extrusion (EMP), injection molding (IMP), and hot pressing (HPMP). The binary pictures of composites fracture surface for EMP, IMP, and HPMP were diverse (Figure 8), and the surface fractal dimension of the three types were different; EMP composites needed the least potential energy to change the structure, the interfacial adhesion of EMP composites was the best, while the HPMP composite was the worst [72].

The fractal dimension of AFM images describing the characteristic structure of z-scale factors can reflect the fractal characteristics of materials more comprehensively than the fractal dimension obtained by roughness [73]. Gabibov et al. [74] found that the composite structure composed of styrene butadiene rubber (SBR) and epoxy resin ED-20 filled with nanoparticles is a combination of two fractals. The “disturbance” of polymer matrix structure caused by filler particles depends on the concentration and size of the initial filler particles; they found that the fractal dimension of the aggregate surface of the initial filler particles affects the fractal dimension and size of the filler particles; the particle size and aggregation parameters grow; and the fractal dimension of particle aggregates increases.

In summary, the application of the fractal dimension of the z-scale factor characteristic structure in the AFM image can more fully reflect the fractal characteristics of the material surface. This parameter may help better analyze the material properties, which may be a direction that subsequent researchers need to pay attention to.

### 4.3. Relationship between Fractal Dimension and Properties of Polymers and Their Composites in the Packaging Industry

**Figure 9:**
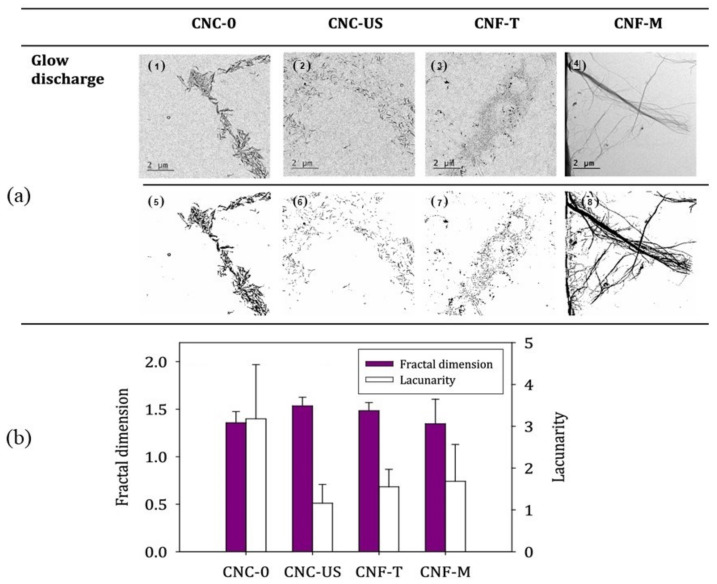
TEM images (**a**) (pretreated through glow discharge (subfigures **1**–**4**) and processed images (subfigures **5**–**8**)) and fractal dimension (**b**) of CNCs and CNFs samples.

With the development of fractal theory and material properties, people are more inclined to figure out the relationship between the fractal dimension and properties of polymers and their composites. In recent years, research on the fractal dimension and properties of packaging paper, natural plant fiber, petroleum-based polymer and their composites, nano materials, and porous materials in the packaging industry mainly comprise the following aspects:

With regard to packaging paper and natural plant fiber, SEM and TEM are usually used to acquire surface morphology, and fractal theory can also be applied for analysis. The surface of food packaging paper was scanned by SEM, and surface porosity was analyzed according to fractal theory [75]. Campano et al. [76] investigated the bulk morphology of CNCs and CNFs by TEM, and quantified the aggregation/dispersion degree and fibrillation degree of CNFs by fractal dimension and porosity, as shown in Figure 9.

The properties of other polymers and their complex materials in the packaging industry was researched by the fractal dimension. Dispersion of reduced graphene oxide within thermoplastic starch/poly (lactic acid) blends was investigated by small-angle X-ray scattering; the formation of a fractal structure led to the significant enhancement of macroscopic properties [77]. In sustainable biocomposites from Nylon 6 and polypropylene blends and biocarbon, angular particles having a high polydispersity and porosity were shown to form pendular and funicular morphologies when compounded in immiscible blends, and their morphology could be distinguished by fractal dimension; the mechanical properties of the composite are better than that of the original [78]. The fractal characteristics of impact fracture surface of bamboo plastic composite with core shell structure were analyzed by the box counting dimension method, the surface fractal dimension was in the range of 2.2075–2.2204, the relationship between the impact strength and the fractal dimension of fracture surface was an approximate power exponent, indicating that the surface fractal dimension of fracture could reflect the impact strength of core-shell structure bamboo plastic composite [79]. Fractal characteristics of the impact strength of polypropylene/polypropylene grafted maleic anhydride/potassium hexatitanate whisker (PP/PP-g-MAH/SPTW) composite packaging materials were studied by box counting method using FractalFox software, the fractal dimension of cross-section was in the range of 1.4165–1.8832, and the impact strength of PP/PP-g-MAH/SPTW was positively correlated with the power exponent of fractal dimension [80]. The fractal dimension of tensile section of polypropylene/fly ash composite filled with the silicon carbide whiskers was computed by the differential box counting method from 1.24 to 1.90, and the logarithm of tensile strength has a linear function relationship with its fractal dimension [81]. Lu et al. [82] found that with the increase of nano AlN content, the microcrack fractal dimension of nano AlN/PTFE composite increases. The larger the fractal dimension of microcracks, the more disordered the distribution of microcracks on the material surface, which is conducive to the formation of a transfer film on the dual surface of the composite material, and helps to improve wear resistance; the greater the fractal dimension of microcracks, the more disordered the distribution of microcracks on the surface of materials, the tensile fracture of materials gradually changes from ductile fracture to brittle fracture, resulting in the decrease of tensile properties of materials. The effect of fractal dimension and branch number on thermal conductivity of Si/Ge nanocomposites with fractal tree network was studied by the molecular dynamics simulation method, the Si/Ge nanocomposites with fractal tree network had greater length and width, fractal dimension and more branch layers than other Si/Ge nanocomposites; the sub interface scattering is stronger, and the fractal tree network is better than the traditional rectangular core structure in reducing the thermal conductivity of nanocomposites [83]. The structural stability and properties of the dispersed filled polymer were analyzed by using the macro structure image processing results based on the texture method and multifractal method [84]. Wu et al. [85] prepared glass bead/polypropylene (GB/PP) composite by the melt blending method, observed the dispersion morphology of GB in the cross-section of the composite by SEM, and calculated the dispersion fractal dimension (Dd) of GB by fractal model. The results demonstrated that Dd can quantitatively characterize the dispersion effect of GB; the larger the Dd, the more uniform the dispersion of GB, the higher the impact strength and toughness of the composites.

For the fractal structure, the second moment of particles in composites is expressed as $<r^{2}\left(t\right)>\propto t^{2\alpha }$, where α is Hurst index, which can reflect the diffusion law of particles. When Hurst index is greater than 0.5, it is part of accelerated diffusion; when it is 0.5, it is free diffusion; when it is less than 0.5, it pertains to secondary diffusion, and diffusion is slow. Qu et al. [86] obtained the diffusion law of particles in fractal structure by numerical simulation and comparison of Hurst index and its influencing factors in the condition that particles cannot enter the matrix and can move in the matrix and gap at the same time. Jelcic et al. [87] found that the average fractal index of the fracture surface of the binary elastomer of impact resistant polystyrene (HIPS) and polystyrene-b-polybutadiene-b-polystyrene block copolymer (SBS) is less than 2, and the fractal results are related to the mechanical properties of the blends and the Hurst index obtained from the time series of processing parameters (e.g., torque and melt pressure). Moreover, for the heterogeneous polymer blends, the processing and mechanical results agree with the fractal characteristics of the fracture surface [88].

Apart from the earlier calculation method of fractal dimension of graphics, the signal fractal dimension calculation method is also applied to the performance analysis of composite materials. Based on the non-stationary AE signals in tensile and bending tests of glass fiber reinforced polymer matrix composites, the Hurst index, the scaling index of DFA analysis method, the minimum covering dimension and the box counting dimension of the AE signal were calculated when matrix cracking, fiber/matrix peeling, delamination, and fiber fracture were occurred; the foregoing indicators can distinguish different failure mechanisms; the AE signal of the product has multifractal behavior [89].

**Figure 10:**
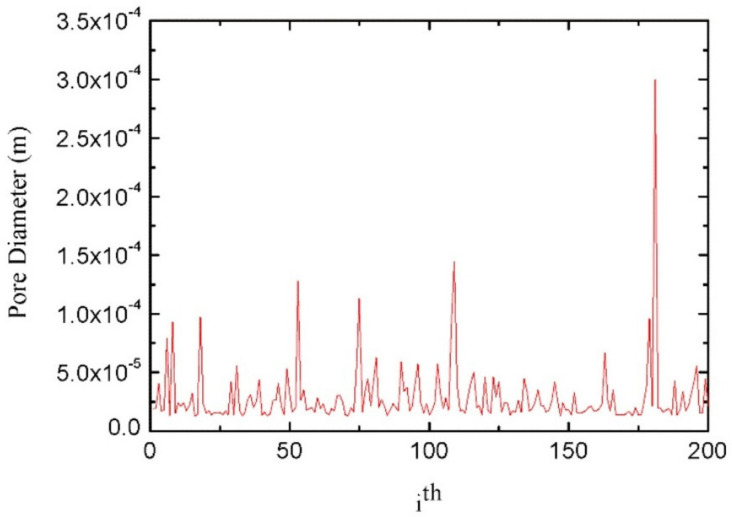
The capillary sizes simulated by Xiao’s Fractal-Monte Carlo simulations with poroity of 70%.

Porous materials with low relative density, large specific strength, and specific surface area have been widely concerned in the field of packaging material. The preparation and properties of porous materials are one of the key focuses in the field of packaging materials. Compared with other packaging materials, the fractal research of porous polymer materials for packaging is more in-depth and detailed, including fractal dimension, mechanical properties of porous materials, adsorption and seepage process, and property relationship. Zhang et al. [90] obtained the pressure (p) and mercury injection (V) of Aramid paper-based materials by MIP, and then calculate the slope of ln(dV/dp)-lnp curve, namely, the fractal dimension. The fractal dimension was positively correlated with the average pore diameter, porosity, and specific surface area of Aramid paper-based materials, but negatively correlated with its tensile index, tear index, and compressive strength. Fractal dimension can be available to study the pore structure, and the advantages and disadvantages of the mechanical and insulation properties of Aramid paper-based materials. Liu et al. [91] established the fractal structure of wetting line by using the internal growth mode of half hole circular arc, and included the accumulation effect of inertial force on the circular arc into Bosanquet’s inertial force imbibition mechanism. The fractal dimension and pore size range of paper coating materials were measured by the FHH model of multi-layer adsorption of gas molecules in porous media. The fractal dimension and pore size range are reasonable and normal, and the fluid inertia force has a positive effect on the rapid imbibition in mesoporous and nano channels. Not only the structural parameters of paper coating materials have fractal characteristics, but also the bending properties of capillary tubes in the process of imbibition [92], and the wetting angle of rough surfaces [93]. According to the fractal characteristics of pores in porous media, considering the capillary pressure, gravity, and fracture from spontaneous absorption of wetting liquid into saturated porous media of gas (especially the mechanism of fracture enhanced spontaneous imbibition), a fractal model of fracture index for single-sided fracture is established, which could study the relationship between the cumulative mass and the contact area, pore fractal dimension, curvature, maximum pore size, porosity, liquid density and viscosity, surface tension, contact angle, crack height and inclination angle of wetting liquid [94]. The probability model of the Kozeny-Carman constant of fibrous porous media is erected by the fractal Monte Carlo technique, as seen in Figure 10. The model is a function of structural parameters of fibrous porous media, including porosity, pore size, fiber diameter, curvature, and area fractal dimension, which can represent other transmission features of fluid in fibrous porous media [95]. The improved fractal structure of Sierpiński carpet can reconstruct the porous media model, which is applied to simulate the fluid seepage process under the potential difference through the random walk process of particles with certain directivity in the pore channel. The quantitative characterization of the complex pore structure of porous media is realized by the directional random walk fractal spectrum dimension; the larger the fractal dimension of random walk in the direction, the greater the seepage performance in this direction, namely, the better the connectivity of pore channels; nevertheless, there is a huge gap between the simulation results and the actual three-dimensional flow process of porous media [96].

The above research on porous materials is based on the classical mechanical theory. The continuity assumption is applicative in many practical applications, such as space, air flow and water flow. Even so, if molecules diffuse in water, the water will become intermittent; in the framework of a continuum hypothesis, the movement of molecules will become completely unpredictable [97]. Fractal dynamics takes place on a very small-time scale, which is a discontinuous system. Fractal calculus [98,99] can be used to describe the molecular motion in this term. Fractal calculus is a comparatively new concept, which is able to productively handle fractal dynamics by replacing continuous time with fractal time [100,101]. Fractal calculus can effectively simulate various phenomena in porous media or hierarchical structure, and reveal the hidden mechanism that cannot be found in continuum mechanics [102,103].

In summary, for many packaging materials’ preparation and performance analysis process, a new processing could be first using SEM, TEM, SAXS, and other imaging technologies to obtain object images, or utilizing mercury intrusion method to extract the fractal features of porous materials, and then employing fractal dimension calculation methods, such as box counting dimension, multifractal method, Hurst index, and signal fractal dimension calculation method, finally, establishing the relationship between fractal dimension and material properties. This method may be a reference for other materials that have not been analyzed by fractal theory, and may become a new and better performance analysis method. With the deepening of fractal dynamics research, the establishment of the relationship between fractal dimension and material properties is expected to provide a new method for predicting material properties on a small scale.

## 5. Conclusions

At present, fractal theory has been applied to a large number of packaging material property research, such as metal alloys and ceramics, polymer and its composites, but there are also many packaging material performance analyses without fractal theory.

In this paper, the fractal analysis methods applied to inorganic materials such as metal alloys and ceramics, polymers, and their composites are reviewed from the aspects of fractal feature extraction and fractal dimension calculation. Through the fractal dimension model of packaging materials, the relationship between the fractal characteristic parameters of packaging materials and their properties was analyzed. The fractal analysis method has become a new method for qualitative and quantitative analysis of packaging material preparation and property research; furthermore, it may be more accurate and effective to employ multi-fractal analysis to evaluate and predict the properties of packaging materials. Research on the relationship between fractal dimension and packaging material preparation and property has been one of the focuses of the field of packaging material property research.

However, due to the abstract fractal theory, the research results are only obtained based on the phenomenological theory, and do not go deep into the mechanism. Therefore, the important direction of fractal theory about the aspect of packaging material in the future mainly includes the following: establishing a fractal analysis method on a very small-time scale to analyze the properties of hierarchical structure or discontinuous structure; building a model which unifies the microstructure and material performance with fractal theory to explain the mechanism of change in packaging material property, and analyzing the preparation process of packaging materials by fractal dynamics.

## Data Availability

Not applicable.
